# Tumor necrosis factor-alpha gene −308G > A polymorphism alters the risk of hepatocellular carcinoma in a Han Chinese population

**DOI:** 10.1186/s13000-014-0199-3

**Published:** 2014-11-25

**Authors:** Hua Feng, Jing-hua Kuai, Ming-yan Zhang, Guang-chuan Wang, Yong-jun Shi, Jun-yong Zhang

**Affiliations:** Digestive Disease Department, Shandong Provincial Hospital, Affliated to Shandong University, Jinan, Shandong China; Digestive Disease Department, Qi Lu Hospital of ShanDong University (QingDao), QingDao, Shandong China; Degestive Disease Department, Shandong Provincial Hospital, Num 324, JingWu Wei Qi Road, Jinan, PR China

**Keywords:** Hepatocellular carcinoma, TNF-α, Genetic variant, Susceptibility, Risk

## Abstract

**Background:**

The aim of the present study was to evaluate the influence of tumor necrosis factor-alpha (TNF-α) −308 G > A polymorphism on hepatocellular carcinoma (HCC) risk.

**Methods:**

The present case–control study was conducted in a Han Chinese population consisting of 753 HCC patients and 760 controls from May 2010 to March 2013. The −308 TNF-a promoter polymorphisms were detected. Conditional logistic regression was performed to analyze the association between TNF-α −308 G > A polymorphism and the risk of HCC, which were estimated by odds ratios (ORs) and their 95% confidence intervals (95% CIs).

**Results:**

The genotypic frequencies in the cases were not similar to that of the controls, differences being statistically significant (*P* = 0.002). Using the GG genotype as the reference genotype, AA was significantly associated with increased risk of HCC (adjusted OR = 5.12, 95% CI = 2.31–7.82). Similarly, AG + AA genotype showed 5.59-fold increased HCC risk in a dominant model. Furthermore, we found A allele was significantly associated with increased risk of HCC, compared with G allele (OR = 4.18, 95% CI = 1.76–6.97).

**Conclusion:**

The present study showed that TNF-α −308 G > A polymorphism was associated with increased HCC risk in a Han Chinese population. Further prospective studies on large and different ethnic populations will be necessary to confirm our findings and elucidate the underlying molecular mechanism for the development of HCC.

**Virtual Slides:**

The virtual slide(s) for this article can be found here: http://www.diagnosticpathology.diagnomx.eu/vs/13000_2014_199

## Background

Hepatocellular carcinoma (HCC) is one of the common malignant tumors globally, which is the fifth most prevalent cancer and the third cause of cancer-related deaths worldwide [1]. Approximately 650,000 cases die from HCC each year, and >75% of these cases occur in the Asia-Pacific region [2]. It is indicated that China has a very high incidence with about 55% of annual new cases of HCC worldwide [3]. HCC has been one of the most common causes of cancer-related deaths in China. Although chronic hepatitis B virus (HBV) and hepatitis C virus (HCV) infections, aflatoxin B1, alcohol and nonalcoholic steatohepatitis are regarded as the main carcinogenic mechanism, only a few of these patients with these risk factors develop HCC during their lifetime, suggesting the etiology of HCC is not well clarified [4]. Thus some genetic factors may contribute to the carcinogenic mechanism.

Tumor necrosis factor-alpha (TNF-α) encodes a pro-inflammatory cytokine that is secreted primarily by macrophages and plays critical roles in the pathogenesis of inflammatory autoimmune and malignant diseases. The TNF-α protein induces the expression of adhesion molecules, facilitating the invasion of metastatic tumor cells [5], and high levels of endogenous TNF-α have been observed in the blood of some cancer patients. Several polymorphisms in the promoter region of TNF-α have been associated with different TNF-α expression levels [6]. Of these, the TNF-α −308 G > A polymorphism is the best studied. It involves the substitution of a guanine (G) by an adenine (A) and is associated with an increase in TNF-α expression levels [7]. TNF-α −308G > A polymorphism has been reported to alter the risk of several types of cancers, such as breast cancer, lung cancer, non-Hodgkin lymphomas, and prostate cancer [8-11]. However, the association between TNF-α −308 G > A polymorphism and risk of HCC is controversial [12-15]. The present case–control study was performed to assess the association of HCC risk and TNF-α −308 G > A polymorphism in a Han Chinese population.

## Methods

### Study population

The present case–control study consisted of 753 HCC patients and 760 cancer-free controls from May 2010 to March 2013 at the Digestive Disease Department, Shandong Provincial Hospital Affliated to Shandong University. All subjects were unrelated Han Chinese living in China. Health subjects were randomly selected from health screening program participants to exclude those with a history of cancer and other medical diseases. All HCC patients were diagnosed on the basis of histology or the combination of typical radiological findings of HCC, and underwent surgery in Shandong Provincial Hospital Affliated to Shandong University. The tumor stage of HCC cases was evaluated on the basis of the tumor-nodule-metastasis (TNM) classification system. The HBsAg and anti-hepatitis C virus (HCV) antibody were tested by microparticle enzyme immunoassays using commercial assay kits, which were used to determine the infection status of hepatitis B or hepatitis C. Clinical characteristics data as well as related risk factors, including gender, age, smoking status, drinking status, serum a-FP levels, family history of HCC and HBV, HCV serological markers, were summarized (Table 1).The present study was approved by the Medical Ethics Committee of Shandong Provincial Hospital Affliated to Shandong University, and informed consent was obtained from all participants.

**Table 1:** **Clinical characteristics of hepatocellular carcinoma cases and healthy controls**

**Characteristics**	**Cases (n)**	**%**	**Controls (n)**	**%**	**P-value**
Number	753	49.8	760	50.2	
Gender					
Male	467	62.0	450	59.2	0.59
Female	286	38.0	310	40.8	
Age (years)					
<55	307	40.4	303	39.8	0.45
≥55	446	59.6	457	60.2	
Alcohol drinking					
Yes	713	94.7	711	93.6	0.33
No	40	5.3	49	6.4	
Tobacco smoking					
Yes	719	95.5	701	92.2	0.31
No	34	4.5	59	7.8	
HBsAg					
+	311	41.3	-		
-	442	58.7	-		
Anti-HCV					
+	241	32.0	-		
-	512	68.0	-		
Serum a-FP levels					
<400 ng/ml	275	36.5	-		
>400 ng/ml	478	63.5	-		
Family history of HCC					
Yes	119	15.8	-		
No	634	84.2	-		

### DNA extraction

A 5 mL sample of venous blood was collected from each subject into a test tube containing EDTA as anticoagulant. Genomic deoxyribonucleic acid (DNA) was extracted from 2 mL of peripheral blood by the standard method with proteinase K digestion followed by phenol–chloroform extraction. After ethanol precipitation, the DNA was dissolved in double distilled water and frozen at −20°C until use.

### Polymorphism genotyping

The −308 TNF-a promoter polymorphisms were determined by method previously described [16]. Amplification was performed in GeneAmp PCR System 9700 termocycler (Applied Biosystems, Singapore) with 100 ng of genomic DNA, 25 pmol of each primer, 200 μM total dNTP, 1.5 mM MgCI_2_, 1× PCR buffer and 2.5 U Taq DNA polymerase (Promega, Madison, WI, USA). The following cycling conditions were used: 95°C for 5 min, followed by 35 cycles of 94°C for 60 s, 58°C for 30 s and 72°C for 60 s, with a final extension at 72°C for 10 min. Restriction enzyme digestion with NcoI (Promega, Madison, WI, USA) of the PCR product was carried out overnight and analyzed on a 3% agarose gel. DNA products were visualized by ethidium bromide staining. The -308G showed two fragments (homozygous for the allele -308G), while its homologue -308A was undigested and resulted in a single band (homozygous for allele -308A, lacking NcoI site). The presence of all three fragments defined heterozygotic individuals.

### Statistical analysis

All statistical analyses were performed using the Statistical Package for Social Science software version 18.0 (SPSS Inc., Chicago, IL, USA). Continuous variables were reported as means ± standard deviation (SD), and categorical variables were reported as frequencies (N) and percentages (%). The χ^2^ test was used to assess differences between cases and controls with regard to clinical characteristics. A goodness-of-fit χ^2^ test was used to evaluate the Hardy-Weinberg equilibriums in controls. Conditional logistic regression was performed to analyze the association between TNF-α −308 G > A polymorphism and the risk of HCC, which were estimated by odds ratios (ORs) and their 95% confidence intervals (95% CIs). The significance levels of all tests were set at *P* <0.05.

## Results

### Clinical characteristics of cases and controls

In the present case–control study, a total of 1,513 Chinese Han subjects were enrolled, which consisting of 753 HCC patients and 760 cancer-free controls. Table 1 showed the general characteristics of the studied subjects. There were 467 males and 286 females in the HCC group, and 450 males and 310 females in the control group. The mean age of HCC patients was 53.3 years, and mean age of cancer-free controls was 52.9 years. There were no significant differences between HCC cases and cancer-free controls with regarded to gender and age distribution. Besides, for other general characteristics, such as alcohol drinking, tobacco smoking, there were no significant differences between HCC cases and cancer-free controls (all *P* >0.05, shown in Table 1).

### Genotypic frequencies of TNF-α −308 G > A in cases and controls

The observed genotype distribution in the controls did not differ from those expected from Hardy–Weinberg equilibrium (*P* > 0.05). Compared to healthy controls, patients with HCC had lower frequency of GG genotype (81.1% vs. 96.4%) and a higher frequency of AG (13.0% vs. 3.2%). Homozygous AA genotype was found in 44 HCC patients and in 2 of controls. Thus, genotypic frequencies in the cases were not similar to that of the controls, differences being statistically significant (*P* = 0.002, shown in Table 2).

**Table 2:** **Distribution of TNF-α −308 G > A genotypes in cases and controls**

**TNF-α −308 G > A**	**Cases**	**%**	**Controls**	**%**	**P value**
GG	611	81.1	733	96.4	0.002
AG	98	13.0	25	3.2	
AA	44	5.9	2	0.4	

### The association of TNF-α −308G > A polymorphism with HCC risk

We further analyzed the effects of the tested genotypes under different genetic models (shown in Table 3). Using the GG genotype as the reference genotype, AA was significantly associated with increased risk of HCC (adjusted OR = 5.12, 95% CI = 2.31–7.82). Similarly, AG + AA genotype showed 5.59-fold increased HCC risk in a dominant model. Furthermore, we found A allele was significantly associated with increased risk of HCC, compared with G allele (OR = 4.18, 95% CI = 1.76–6.97).

**Table 3:** **The association of TNF-α −308G > A polymorphism with hepatocellular carcinoma risk**

**TNF-α −308 G > A polymorphism**	**HCC patients**	**Controls**	**OR (95% CI)** ^**1**^	**P value**
General genotype				
GG	611	733	1.00 (Reference)	
AG	98	25	2.12 (1.25–3.46)	0.02
AA	44	2	5.12 (2.31–7.82)	0.003
Dominant genotype				
GG	611	733	1.00 (Reference)	
AG + AA	142	27	5.59 (2.41–8.02)	<0.001
Recessive genotype				
AG + GG	709	758	1.00 (Reference)	
AA	44	2	1.98 (0.95–3.27)	0.06
Allele frequency				
G	1,320	1,491	1.00 (Reference)	0.006
A	186	29	4.18 (1.76–6.97)	

^1^ Adjusted for sex, age, smoking status, and drinking status.

## Discussion

Advances in molecular and genetic epidemiology have increased our knowledge of the mechanisms underlying hepatocarcinogenesis and the relationships between susceptibility and individual genetic variations [17]. Based on the genetic information, we determine the disease etiology in terms of genetic determinants to be used for identifying the high-risk individuals and perform targeting therapy to the individual’s genetic make-up.

TNF-α is a member of the TNF/TNFR cytokine superfamily, and is an intercellular communicating molecule involved in building transient or long-lasting multicellular structures [18]. It interacts with receptors TNFR1 and TNFR2, which participate in cellular signal transduction pathways [19]. TNF-α plays an important role in the regulation of cell differentiation, proliferation and death as well as in inflammation and the innate and adaptive immune response. It has also been implicated in a wide variety of human diseases. The presence of DNA sequence variations in the regulatory region might interfere with transcription of the TNF gene, influencing the circulating level of TNF and thus increasing susceptibility to human diseases, such as cancer. The TNF enhancer polymorphism has been implicated in several diseases, and the TNF-a −308 polymorphism has been described as the most important TNF polymorphism in human disease susceptibility. The significance of these polymorphisms reflects their possible influence on the transcription of the TNF gene. TNF-α −308 G > A polymorphism involves the substitution of a guanine (G) by an adenine (A) and is associated with an increase in TNF-α expression levels [7]. TNF-α −308G > A polymorphism has been reported to alter the risk of several types of cancers, such as breast cancer, lung cancer, non-Hodgkin lymphomas, and prostate cancer [8-11]. For example, Jin et al. found that the TNF- α −308G > A polymorphism was not associated with breast cancer risk in the overall population but that the A allele might be a protective factor for breast cancer in postmenopausal women, and the AA genotype might be a breast cancer risk factor in premenopausal women [8]. In Ma et al’ study, a significantly increased prostate cancer risk was found to be associated with the TNF-α-308 G > A polymorphism (AA + AG vs. GG: OR = 1.531, 95% CI = 1.093–2.145; *P* = 0.013; AG vs. GG: OR = 1.477, 95% CI = 1.047–2.085; *P* = 0.026). Their results suggested that the TNF-α-308 G > A polymorphism might significantly contribute to prostate cancer susceptibility [11]. However, the association between TNF-α −308 G > A polymorphism and risk of HCC is controversial [12-15]. The present case–control study was performed to assess the association of HCC risk and TNF-α −308 G > A polymorphism in a Han Chinese population. We found that the genotypic frequencies in the cases were not similar to that of the controls. We then analyzed the effects of the tested genotypes under different genetic models. Using the GG genotype as the reference genotype, AA was significantly associated with increased risk of HCC. Similarly, AG + AA genotype showed 5.59-fold increased HCC risk in a dominant model. Furthermore, we found A allele was significantly associated with increased risk of HCC, compared with G allele. Our results were consistent with the findings previously reported by Heneghan et al. and Ho et al. [20,21], but different from Chen et al’s study [22].

## Conclusions

In conclusion, the present study showed that TNF-α −308 G > A polymorphism was associated with increased HCC risk in a Han Chinese population. Further prospective studies on large and different ethnic populations will be necessary to confirm our findings and elucidate the underlying molecular mechanism for the development of HCC.
